# Exploring the bidirectional within-subject relationship between sleep and affective wellbeing: Insights from an intensive longitudinal study

**DOI:** 10.1016/j.ijchp.2025.100648

**Published:** 2025-11-13

**Authors:** Justin Hachenberger, Maia ten Brink, Denny Kerkhoff, Sebastian Baron, Manuel Schabus, Sakari Lemola

**Affiliations:** a Department of Psychology, Faculty of Psychology and Sports Science, Bielefeld University, Germany; b Columbia University Irving Medical Center, Center for Behavioral Cardiovascular Health, Department of Medicine, New York, NY, United States; c Department of Artificial Intelligence and Human Interfaces (AIHI), Paris-Lodron University of Salzburg, Salzburg, Austria; d Laboratory for Sleep, Cognition and Consciousness Research, Department of Psychology, Centre for Cognitive Neuroscience Salzburg (CCNS), Paris-Lodron University of Salzburg, Salzburg, Austria

**Keywords:** Sleep staging, Heart rate variability, Intensive longitudinal data, Affect, Mood

## Abstract

This study aimed to investigate the bidirectional relationships between affective wellbeing and sleep, and to explore the moderating effects of depressive and insomnia symptoms. Data from two studies, involving 178 participants in total aged 18–29 years, were analyzed. Over 14 days, participants completed daily surveys assessing affective wellbeing and wore ECG chest belts for heart rate variability monitoring to derive objective sleep indices. Multilevel models were used to examine within-subject associations between affective states and subsequent sleep, and vice versa, while considering depressive and insomnia symptoms as moderators. Higher positive affect than usual in the evening was associated with shorter total sleep time, shorter rapid eye movement sleep, and lower number of awakenings and stage shifts than usual in the following night. Regarding sleep predicting morning’s affective wellbeing, longer slow-wave sleep was linked to higher positive affect than usual, while longer rapid eye movement sleep and higher sleep efficiency than usual predicted lower negative affect than usual. Also, higher subjective sleep quality than usual was associated with higher positive and lower negative affect than usual the next morning. No evidence for moderation effects of insomnia and depressive symptoms for the bidirectional within-subject associations between sleep and affect was found. The findings underscore the complex interplay between sleep and affective wellbeing, particularly highlighting that the subjective perception of one’s sleep appears to be relevant for the next morning’s affective states.

## Introduction

Sleep plays an important role for affective wellbeing (e.g., Lemola et al., 2013; Ong et al., 2017; Tang et al., 2017; Hickman et al., 2024). There is abundant evidence that after nights with poor subjective sleep quality (SSQ) levels of positive affect are reduced while levels of negative affect and symptoms of depression and anxiety are increased (Das-Friebel et al., 2020; Hachenberger et al., 2023; Konjarski et al., 2018; Lenneis et al., 2024; Ong et al., 2017; Hickman et al., 2024; ten Brink et al., 2022). Similarly, studies applying experimental sleep deprivation or restriction show a subsequent reduction of affective wellbeing (see Palmer et al., 2024 for a meta-analysis). This aligns with findings of a recent systematic review of the daily relationship of sleep and affect in naturalistic settings (Hickman et al., 2024) which revealed strong and consistent links between objective total sleep time (TST) with subsequent affective wellbeing. However, for other objective sleep indices, such as sleep efficiency (SE), sleep-onset latency (SOL), wake time after sleep onset (WASO) and number of awakenings (NOA), the evidence regarding their relationship to affective wellbeing is limited or inconsistent. While most studies have focused on the role of SSQ, TST, SOL, sleep continuity (SE, WASO, NOA), there are three lines of research which also considered the association between sleep architecture and affective wellbeing.

A first line of research has employed cross-sectional designs and focused on comparing sleep architecture in individuals with major depressive disorder (MDD) with healthy controls, where individuals with MDD showed increased amounts of rapid eye movement sleep (REM), decreased REM latency, and decreased deep sleep (N3) (Palagini et al., 2013; Baglioni et al., 2016). An interpretation of these findings is that REM is involved in affective reprocessing of negative experiences in MDD. However, this process is thought to be deficient in patients with depressive disorders due to an instability of REM (Riemann et al., 2012). In turn, insufficient or incomplete processing of affective experiences due to disrupted REM is postulated to lead to a rebound effect on subsequent nights of sleep, manifesting as increased REM pressure involving decreased REM onset latency after sleep onset, increased REM density during REM periods (i.e., increased number of rapid eye movements), increased overall amount of REM per night (Riemann et al., 2012). Thus, according to this interpretation, the brain may become stuck in the attempt of reprocessing of negative memories during REM which is predisposed to fail due to instability of REM. Another interpretation focuses on a different aspect of sleep architecture, namely, the decreased amount of slow-wave sleep (SWS) in MDD, which might lead to insufficient recovery over-night considering that SWS is supposed to be the most restorative sleep stage (Cudney et al., 2022).

The second line of research, which mainly involves experimental work in non-clinical samples, has focused on presenting individuals with emotionally distressing or embarrassing events and tested (1) which aspects of sleep architecture were increased thereafter and (2) whether the duration or relative amount of certain sleep stages was associated with memory performance for the events and/or whether the affective response upon re-exposure to cues reminding of these events the next morning was diminished after increased amounts of REM sleep or SWS (Walker & van der Helm, 2009). Prominently, the Sleep to Forget/Sleep to Remember (SFSR; Walker & van der Helm, 2009) theory suggests that REM sleep facilitates consolidation of declarative memory, while at the same time it decreases the affective tone associated with it. While the SFSR theory produced ample support regarding the consolidation of declarative memory (Cunningham et al., 2014; Hu et al., 2006; Payne & Kensinger, 2010; Wiesner et al., 2015), there is also conflicting evidence, particularly regarding the notion that REM sleep is associated with a decrease of the associated affective tone (see Werner et al., 2015, for an overview). Acknowledging these conflicting study results, Baran et al. (2012) and Pace-Schott et al. (2012) proposed an alternative interpretation of the role of REM sleep, which suggests that REM sleep consolidates the emotional salience of memories, which makes these memories more readily available the next day. This may be adaptive in evolutionary terms, but also leads to an increase in negative affect, at least in the short term. This notion has also found some empirical support (Werner et al., 2021).

A third line of research analyzed affective wellbeing on the state level during the days before and after polysomnographic (PSG) assessment of sleep. However, to date we are aware of only four studies adopting such a design. Zhang and colleagues (2023) found that higher positive affect during the day may be associated with less fragmented REM sleep in the subsequent night. As a major shortcoming, this study only employed a single night design, which does not allow disentangling within-subject from between-subject variation. Messman et al. (2021) also employed an intensive longitudinal design across seven days with ambulatory single-channel electroencephalography (EEG) to assess sleep. They found that higher morning negative affect than usual was followed by longer REM in the subsequent night. SWS and REM, however, were not associated with subsequent morning affective states. Zapalac et al. (2024) employed an intensive longitudinal design to monitor 65 young adults over an average of 35 days and nights, which allowed for separate within-subject analyses testing associations between sleep architecture and affect ratings the next morning. They found that a longer REM onset-latency was associated with lower stress in the morning, while no evidence for an association between the REM to non-REM sleep ratio (REM/NREM) with morning mood was found. In the study of Boon et al. (2025), 33 participants wore an EEG headband during sleep for 15 days, and home-based polysomnography was also employed on three days. No significant associations were found between the EEG headband-based sleep measures (time in REM and SWS, REM fragmentation) and positive or negative affect. However, higher negative affect in the evening was associated with higher REM fragmentation measured by PSG the following night.

Evidence suggests that between-subject variability is often produced by different mechanisms than variability within subjects over time (Fisher et al., 2018; Hamaker, 2012; Hamaker & Wichers, 2017). To disentangle within- and between-subject variability, it is necessary to assess the variables of interest over multiple nights in an intensive longitudinal design. As PSG poses a considerable burden for participants, PSG studies over more than two consecutive nights are rare. Recently, procedures have been proposed that allow for reliable detection of sleep stages (i.e., wake, N1 & N2, N3, REM) based on the interbeat intervals (IBI) of the heart rate variability (HRV; Topalidis et al., 2023a, 2023b) which can be monitored unobtrusively over longer time periods. To our knowledge, the study by Zapalac et al. (2024) is the only one to date employing such a procedure based on HR/HRV to study within-subject associations between sleep architecture and affective wellbeing, while controlling for between-level factors (Zapalac et al., 2024).

Given the limited and mixed findings on the within-person bidirectional relationships between naturalistic affective wellbeing in daily life and objectively assessed sleep characteristics (ten Brink et al., 2022; Konjarski et al., 2018; Hickman et al., 2024), specifically concerning sleep architecture, the main aim of the present study is to identify the key aspects of sleep that bidirectionally relate to fluctuations in affective wellbeing in individuals’ daily lives. The present study uses sleep indices that are derived from HRV-IBI which was measured during a 14-day ambulatory assessment study to examine the following research questions: How is affective wellbeing in the evening related to subjective and objective sleep indicators in the subsequent night (RQ1)? How are subjective and objective sleep indicators related to affective wellbeing the next morning (RQ2)? Are the relationships investigated in RQ1 and RQ2 moderated by baseline levels of depressive and insomnia symptoms (RQ3)?

As a subjective sleep indicator, we investigated SSQ, while as objective sleep indicators, TST, SOL, sleep continuity measures (SE, WASO, NOA), sleep architecture (SWS, REM) as well as fragmented SWS and REM as indicated by stage shifts between SWS, REM, and wake state (number of stage shifts; NSS) were investigated. Due to (a) an ambiguous picture that emergers from the literature regarding within-subject associations between affective wellbeing and subsequent sleep, and (b) between sleep continuity (SE, SOL, WASO, NOA) measures and subsequent affective wellbeing, as well as (c) the lack of research on within-subject associations of sleep architecture (SWS, REM) and affective wellbeing in an intensive longitudinal design, we decided to set up our study exploratively. For SSQ only, we hypothesized that it would be associated with higher positive affect and lower negative affect the next day.

## Methods

### Design and procedure

Data from two studies using an experience sampling methodology (ESM) were used to answer the research questions of the present study. We pooled data from two studies to increase the statistical power for the analyses in this manuscript. Both studies are identical in their design with regard to duration, sampling frequency, and overall procedures. The first study could be seen as a pilot study in which we tested the implementation of the electrocardiography sensors for the first time. The second study was the main study, in which only a few changes were made to some items, but these did not concern the constructs or items analysed here.

Both studies were approved by the Ethics Committee of Bielefeld University. Data was collected between May and July 2023 for Study 1 and between November and December 2023 for Study 2. When registering for the study, participants were informed about the procedure and conditions of study participation as well as their rights (e.g., regarding data protection and withdrawal from the study). All participants provided informed consent and confirmed that they met the following inclusion criteria: (a) age 18 to 29 years, (b) being fluent in German, and (c) having a smartphone with an Android operating system available for the duration of the study (participants could receive a lab phone for participation). For Study 2, participants additionally had to confirm that they had not participated in Study 1.

Data collection lasted 15 days for each batch in both studies. On the first day, participants completed a baseline assessment. ESM surveys started on the second day using the movisensXS app (movisens GmbH, Karlsruhe, Germany). Participants were asked to complete short surveys seven times per day. Additionally, participants wore accelerometers (wGT3X-BT®, ActiGraph, LLC, Pensacola, Florida, USA) on their non-dominant wrist during the ESM period and an electrocardiography (ECG) chest belt (Polar® H10, Polar Electro Oy, Kempele, Finland). Participants were informed that they could take off the sensors if a situation required it (e.g., when showering). However, participants were encouraged to wear it as much as possible.

Participants were able to manually start the first survey of each day and were asked to do so as soon as possible after waking up. If participants did not start the first survey manually, they received a reminder prompt between 8:30am and 9:00am on weekdays and between 9:30am and 10:00am on weekends. For the other six surveys during the day, prompts were sent out at random times. However, only the first and last survey of each day (i.e., the morning and evening surveys) were investigated in the present study. A survey would be automatically marked as missing when participants did not respond. Participants were instructed to ignore prompts in situations that could cause danger to themselves or others (e.g., while driving). In each survey, participants reported about their momentary affect, self-rated cognitive performance, physical activity, self-regulation, and contextual information. In the first survey of each day, participants also reported on their sleep the previous night, while the last survey of each day also included diary-like questions about physical activity and daily contextual information. Only the first questionnaire of each day was considered in the present study. The total number of items answered per survey varied between 29 and 59, as some items were only presented conditionally based on previous answers. Participants took an average of 2.7 min to complete the surveys.

### Participants

A total of 71 participants took part in Study 1 and 137 participants in Study 2, resulting in 208 participants in the pooled data set. Of these, 22 participants were excluded because complete data were available for less than three nights and eight participants were excluded because they did not report their gender or reported their gender as diverse, so that sleep based on ECG data could not be analyzed. To test systematic differences between participants that were excluded because of missing data and the final sample, we compared both groups regarding their PHQ-9 and ISI scores as well as their mean SSQ, positive affect, and negative affect. We only found a significant difference for positive affect, in that the excluded participants showed a higher positive affect (*M* = 61.7) than the final sample (*M* = 52.3; *t*(10.7) = –2.45, *p* < .05).

The final sample consisted of 178 distinct individuals (75.8 % female; 24.2 % male). The mean age was 22.4 years (*SD* = 3.2; range 18–29 years). Participants were mostly students (92.7 %). Although a non-clinical sample was investigated, approximately 42.7 % and 15.7 % could have been categorized as having a subthreshold insomnia and clinical insomnia (as indicated by ISI scores, see section Insomnia Symptoms), respectively. Also, 32.6 % of the sample could have been categorized as having moderate to severe depressive symptoms (as indicated by PHQ-9 scores, see section Depressive Symptoms). No participants were excluded from the sample due to mental or physical health conditions or the use of medication.

Of the 2492 possible nights, participants provided data (complete first survey of each day and valid ECG data for the night) for a total of 1936 nights (77.7 %). On average, complete data of 10.9 nights (*SD* = 3.4) was available per participant and 153 participants provided data for seven nights or more. Separate sample descriptions for both studies are presented in Table 1.

**Table 1:** Demographics and descriptive statistics.

	Study 1 (*N* = 61)	Study 2 (*N* = 117)	Pooled data (*N* = 178)	
	*M* (*SD*) / *n* (%)	*M* (*SD*) / *n* (%)	*M* (*SD*) / *n* (%)	*Observed* Min*–*Max
*Demographics*				
Gender				
Female	49 (80.3)	86 (73.5)	135 (75.8)	–
Male	12 (19.7)	31 (26.5)	43 (24.2)	–
Age [Years]	23.3 (3.2)	21.9 (3.1)	22.4 (3.2)	–
*Baseline*				
Depressive symptoms (PHQ-9) a	8.3 (5.4)	8.2 (4.2)	8.3 (4.6)	0–26
No–mild symptoms	40 (65.6)	80 (68.4)	120 (67.4)	–
Moderate–severe symptoms	21 (34.4)	37 (31.6)	58 (32.6)	–
Insomnia symptoms (ISI) b	8.5 (4.9)	9.5 (5.2)	9.2 (5.1)	0–22
No Insomnia	29 (47.5)	45 (38.5)	74 (41.6)	–
Subthreshold Insomnia	23 (37.7)	53 (45.3)	76 (42.7)	–
Clinical Insomnia	9 (14.8)	19 (16.2)	28 (15.7)	–
*Affect Measures*				
Positive Affect (Morning)	46.0 (13.8)	47.3 (14.3)	46.8 (14.1)	11.9–81.2
Positive Affect (Evening)	53.4 (15.3)	53.7 (13.2)	53.6 (13.9)	17.3–90.3
Negative Affect (Morning)	23.3 (15.4)	21.3 (14.2)	22.0 (14.6)	0.2–79.9
Negative Affect (Evening)	21.1 (14.3)	20.1 (14.0)	20.4 (14.1)	0.0–62.4
*Daily Sleep Measures*				
Nights Available	10.3 (3.0)	11.2 (3.6)	10.9 (3.4)	3–14
Sleep Midpoint [hh:mm]	03:54 (00:42)	04:12 (01:36)	04:06 (01:24)	01:42–05:18
Subjective Sleep Quality	58.3 (15.4)	57.2 (15.2)	57.6 (15.3)	12.0–97.7
Total Sleep Time (TST) [h]	6.1 (0.8)	6.5 (0.8)	6.4 (0.8)	3.7–8.5
Sleep Efficiency (SE) [%]	89.7 (5.4)	90.0 (4.0)	89.9 (4.5)	67.2–97.6
Sleep Onset Latency (SOL) [h]	0.4 (0.2)	0.4 (0.3)	0.4 (0.2)	0.1–2.1
Wake After Sleep Onset (WASO) [h]	0.4 (0.4)	0.3 (0.2)	0.4 (0.3)	0.0–2.5
Number of Awakenings (NOA)	19.7 (5.6)	21.6 (5.8)	20.9 (5.8)	2.0–39.0
Slow Wave Sleep (SWS) [h]	1.3 (0.3)	1.3 (0.3)	1.3 (0.3)	0.4–1.9
Rapid Eye Movement Sleep (REM) [h]	1.4 (0.3)	1.6 (0.2)	1.5 (0.3)	0.7–2.1
Number of Stage Shifts (NSS)	14.9 (2.9)	16.1 (3.0)	15.7 (3.0)	7.0–25.0

*Note*. Daily affect and sleep measures were first averaged for each participant.

a Individuals with scores below 10 were considered as having no to mild symptoms. Individuals with scores of 10 and higher were considered as having moderate to severe symptoms (see Kroenke et al., 2001).

b Individuals with scores below 8, 8 to 14, or 15 and higher on the ISI were considered as having no insomnia symptoms, subthreshold insomnia, or clinical insomnia, respectively (see Bastien et al., 2001).

### Measures and instruments

The instruments used to measure the relevant constructs and variables were identical in both studies and are therefore described together.

#### Time-invariant (baseline) variables

**Depressive Symptoms.** Depressive symptoms were measured at baseline with the Patient Health Questionnaire 9 (PHQ-9; Kroenke et al., 2001; Löwe et al., 2002). The participants indicated on a 4-point scale (0 = not at all, 1 = several days, 2 = more than half the days, 3 = nearly every day) how often nine depressive symptoms occurred in the preceding two weeks. Out of all nine items, a sum score was computed (0–27 points). Individuals with scores below 10 were considered as having no to mild symptoms and individuals with scores of 10 and higher were considered as having moderate to severe symptoms (Kroenke et al., 2001).

**Insomnia Symptoms.** Insomnia symptoms were measured at baseline with the Insomnia Severity Index (ISI; Bastien et al., 2001; Gerber et al., 2016). The ISI is composed of seven items that evaluate the severity of sleep difficulties during the past 2 weeks. Each item is rated on a five-point Likert scale and the sum total score indicates the severity of insomnia. Individuals with scores below 8, 8 to 14, or 15 and higher on the ISI were considered as having no insomnia symptoms, subthreshold insomnia, or clinical insomnia, respectively (Bastien et al., 2001).

#### Time-variant (diary and ECG-based) variables

**Subjective Sleep Quality.** SSQ was assessed in the first questionnaire of each day. The participants were asked to indicate on a visual analog scale how satisfied they were with their sleep the previous night (“How satisfied are you with your sleep last night?”, 0 = *not at all*, 100 = *very much*).

**Objective Sleep Indicators Derived from Heart Rate Variability.** HRV in the form of IBI was continuously measured via an ECG chest belt (Polar® H10, Polar Electro Oy, Kempele, Finland). The IBI time series was trimmed for each night from bedtime to wake-up time which participants reported in their sleep diary entries in the first survey of each day (“When did you turn off the light and go to sleep?” and “What time did you wake up this morning?”). For the remaining IBI time series, an epoch-by-epoch four-class sleep staging was performed (with each epoch comprising 30 s of the recording) using a multi-resolution convolutional neural network (MCNN) as implemented in the sleep^2^ app. The MCNN model used in this paper was developed by Topalidis et al. (2023a, 2023b) and is now used in a mobile app that aims to treat insomnia complaints (https://www.sleep2.com; accessed on 18 September 2024). Accordingly, the MCNN analysis were provided by sleep^2^. Topalidis et al. (2023a, 2023b) showed that their trained MCNN is able to classify sleep into four classes (Wake, Light Sleep, Deep/SWS Sleep, and REM Sleep) with an accuracy of 84 % and reaches a relative agreement of 92 % with human experts. Based on the sleep staging, the following objective sleep indicators were calculated: TST (total duration of sleep within the total sleep period), SE (TST/time in bed x 100), SOL (time from lights out until the first sleep epoch), WASO, NOA (frequency of stage shifts from sleep to wake within the total sleep period), time in SWS and REM sleep, and NSS (frequency of stage shifts from SWS or REM sleep to light sleep or wake). Based on the time of falling asleep and final awakening, the sleep midpoint was calculated, which was used as a covariate.

**Affective Wellbeing**. The measurement of affect was based on Das-Friebel et al. (2020). Positive affect was measured with the items content, enthusiastic, and happy. Negative affect was measured with the items sad, upset, and worried. In each ESM survey, the participants were asked to indicate on a visual analogue scale to what extent they felt each affective state (“How … do you feel at the moment?”, 0 = *not at all*, 100 = *very much*). Mean scores for positive and negative affect were computed with higher scores indicating higher levels of positive and negative affect, respectively.

### Statistical analysis

Pre-processing of the data and all statistical analyses were performed using R (version 4.4.1; R Core Team, 2024). To account for the nested structure of our data, we used multilevel models for all analyses described below. The models were fitted using the lmer-function from R-package *lme4* (Version 1.1–35.3; Bates et al., 2015) with restricted maximum likelihood estimation and full randomization. Only in case of convergence issues, the random slopes were removed. All time-variant (level 1) variables were standardized relative to the individual mean (i.e., within-subject standardized) and all time-invariant (level 2) variables were standardized relative to the sample mean (i.e., grand-mean standardized). We included a random intercept and random slopes for all predictors. Sleep midpoint of the respective night, TST of the preceding night, and a variable indicating the type of day (free day = –1, workday = 1) after the respective night were included as covariates in all models in order to adjust for the effects of sleep timing (i.e., circadian processes) and recovery sleep due to prior sleep loss and/or social forces (i.e., homeostatic processes).

To address RQ1, the affective wellbeing variables (i.e., positive and negative affect scores in the evening/before sleep) were modelled as predictors for the sleep variables in the subsequent night which included the SSQ and objective sleep indicators (TST, SE, SOL, WASO, NOA, SWS, REM, NSS). Each outcome variable as examined in a separate model. In these models, the lagged effect of the respective sleep indicator (i.e., the autocorrelation) was included as a further covariate to control for possible sleep rebound following a night of short or poor sleep. Corresponding model equations (for RQ1–3) are presented in Supplement 1.

To address RQ2, we chose a two-step approach: First, for each morning affective wellbeing variable (i.e. positive and negative affect scores in the morning/after sleep) as the outcome, a model was computed that included all objective sleep indicators as predictors. Second, stepwise backwards elimination was applied by discarding the least statistically significant variables, one by one, until all remaining predictors in the model were significant. SSQ as a predictor for affective wellbeing was investigated in separate models.

To address RQ3, the models described for RQ1 and RQ2 were extended by including the time-invariant (between-level) depressive symptoms and insomnia symptoms as moderators and their cross-level interactions with the level-1 predictors. To address the problem of alpha error accumulation in multiple comparisons, all p-values (90 in total) were adjusted by using false discovery rate (FDR; Benjamini & Hochberg, 1995).

## Results

### Descriptive statistics

The demographic characteristics and descriptive statistics of the baseline questionnaires and daily sleep measures are shown in Table 1.

### Affective wellbeing in the evening predicting sleep in the subsequent night (RQ1)

**Fig. 1:**
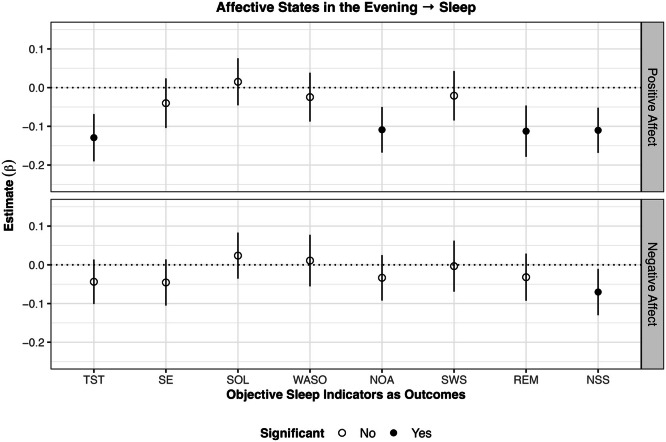
Results of within-subject analyses of affective states in the evening predicting objective sleep in the subsequent night *Note*. SSQ subjective sleep quality; TST total sleep time; SE sleep efficiency; WASO wake after sleep onset; SOL sleep onset latency; NOA number of awakenings; SWS slow wave sleep; REM rapid eye movement sleep; NSS number of stage shifts from SWS/REM to N1/N2/Wake. Lines around the estimate represent 95 % confidence intervals. Higher positive affect in the evening was associated with lower TST, NOA, REM, and NSS in the subsequent night. Lower negative affect in the evening was associated with lower NSS in the subsequent night.

The results of the analyses on evening affective states predicting sleep in the subsequent night are displayed in Fig. 1. Higher than usual positive affect in the evening was associated with lower TST (β = –0.13 *p* < .001), NOA (β = –0.11, *p* < .001), REM (β = –0.11 *p* < .001), and NSS (β = –0.11, *p* < .001) than usual in the subsequent night. Also, higher negative affect than usual was associated with lower NSS than usual (β = –0.07, *p* < .05). No evidence for other significant associations between evening affective states with sleep indicators of the subsequent night were found.

### Sleep predicting affective wellbeing on the subsequent morning (RQ2)

Concerning positive affect, stepwise backwards elimination revealed that longer SWS than usual was associated with higher positive affect the next morning (β = 0.07, *p* < .01). Furthermore, better SSQ than usual (β = 0.30, *p* < .001) was associated with higher positive affect than usual the next morning. The model with only SSQ as the predictor explained 10.6 % of the within-subject variation in morning positive affect, while the model with all objective sleep indicators as predictors explained only 2.2 %.

Concerning negative affect, stepwise backwards elimination revealed that higher SE (β = –0.06, *p* < .05) and longer REM (β = –0.05, *p* < .05) than usual were associated with lower negative affect than usual the next morning. Furthermore, better SSQ than usual (β = –0.19, *p* < .001) was associated with lower negative affect than usual the next morning. The model with only SSQ as the predictor explained 3.6 % of the within-subject variation in morning negative affect, while the model with all objective sleep indicators as predictors explained only 0.8 %.

**Fig. 2:**
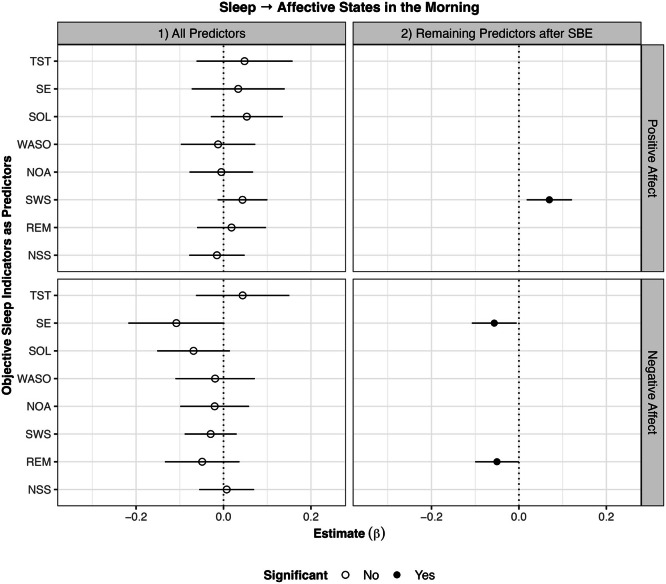
*Results of within-subject analyses of objective sleep indicators predicting affective states in the subsequent morning*. *Note*. SBE stepwise backwards elimination; TST total sleep time; SE sleep efficiency; WASO wake after sleep onset; SOL sleep onset latency; NOA number of awakenings; SWS slow wave sleep; REM rapid eye movement sleep; NSS number of stage shifts from SWS/REM to N1/N2/Wake. Lines around the estimate represent 95 % confidence intervals. Longer SWS than usual was associated with higher positive affect in the morning. Higher SE and longer REM than usual were associated with lower negative affect in the morning.

All results of the analyses on objective sleep indices predicting affective wellbeing on the subsequent day are displayed in Fig. 2.

### Moderation analyses (RQ3)

There was no evidence that any of the associations between sleep indicators and affective wellbeing were moderated by insomnia or depressive symptoms as measured with the ISI and PHQ-9, respectively.

## Discussion

The present study contributes to the growing literature on the dynamics of sleep-affect relationships and offers several insights into this complex interplay. Specifically, we provide novel insights into the role of sleep architecture and continuity for affective wellbeing in an intensive longitudinal design in a sizable sample. There are four key findings of the present research: First, higher evening positive affect than usual was followed by shorter TST, lower NOA, shorter REM, and lower NSS than usual, while higher evening negative affect than usual was only followed by lower NSS than usual. Second, longer SWS and better SSQ than usual were followed by higher levels of morning positive affect than usual. Third, greater SE, longer REM as well as better SSQ than usual were followed by lower levels of morning negative affect than usual. Fourth, no evidence was found that baseline levels of insomnia symptoms or depressive symptoms moderated the bidirectional within-subject associations between affect and sleep. All of these significant within-subject lagged temporal associations between objective sleep indicators and affective wellbeing had only small effect sizes and explained considerably less variation in affect than SSQ.

### Affective wellbeing predicting sleep in the subsequent night

The most notable finding was that higher evening positive affect than usual was associated with shorter TST than usual that night. This finding stands in contrast to previous research, which showed that positive affect is rather associated with longer TST (Hickman et al., 2024; Konjarski et al., 2018). One potential explanation is that high levels of positive arousal in the evening might delay sleep onset or reduce sleep need. Similar effects of high emotional arousal have been observed in relation to sleep (Kalmbach et al., 2020). Both positive and negative heightened affective states, in particular those associated with higher arousal, could interfere with relaxation and sleep initiation. This could reflect an interaction where the timing of positive affect, particularly in the evening, influences sleep differently closer to bedtime than earlier in the day. Regarding the absence of evidence for a link between negative affect and subsequent TST, it could be that it is not overall negative affect, but rather specific aspects of negative affect associated with higher arousal (e.g., as investigated in Werner et al., 2025 or ten Brink et al., 2023) that may be relevant for the TST. Future research should examine the role of individual negative affect items and components in this regard more closely.

The finding that higher positive affect than usual was followed by less REM than usual aligns to some degree with findings by Messman et al. (2021) who observed that higher morning negative affect was followed by longer REM in the subsequent night.

Higher evening positive and negative affect than usual were followed by lower NSS than usual and higher evening positive affect was also associated with less NOA, suggesting reduced sleep fragmentation when experiencing more positive emotions in the evening (see e.g., Pesonen et al., 2019). The result that more negative affect was associated with lower NSS is rather surprising, as negative emotions have previously been linked to increased sleep fragmentation (Vandekerckhove et al., 2011).

Interestingly, besides the association with NSS, no significant associations were found between negative affect and sleep outcomes the following night, which is inconsistent with the intuitive notion and wealth of research linking stress and negative emotions to poorer sleep (Lo Martire et al., 2020; Yap et al., 2020; Newman et al., 2022). This absence of evidence for link may suggest that transient negative affect throughout the day does not significantly disturb sleep when studied on a daily level, supporting the idea that chronic emotional states as with major depressive disorders for example (Palagini et al., 2013; Baglioni et al., 2016) might be more detrimental to sleep than day-to-day fluctuations.

The moderation analyses revealed no significant interactions of affect with baseline levels of insomnia or depressive symptoms, indicating that there is no evidence of these factors influencing how affective wellbeing predicted sleep outcomes on a within-subject level. This is somewhat unexpected given that individuals with major depressive disorder or insomnia are thought to be more vulnerable to emotional disturbances and sleep disruptions (Vargas et al., 2015). However, even though a substantial proportion of participants reported moderate to severe insomnia and depressive symptoms at baseline, the current study did not investigate a clinical sample and did not ask for specific diagnoses which may explain the lack of significant moderation effects.

### Sleep predicting affective wellbeing on the subsequent day

Consistent with previous research, several sleep indices were found to predict next morning’s affective wellbeing. For positive affect, a night with longer SWS than usual was associated with higher positive affect than usual. For negative affect, a night with higher SE and longer REM than usual was associated with lower negative affect than usual.

Our observation that longer than usual SWS was associated with higher than usual positive affect is a novel finding that has not yet been reported in the relatively small number of studies in the naturalistic setting. Traditionally, REM sleep has been associated with emotional processing (Hobson et al., 1998; Konjarski et al., 2018; Walker, 2009), but there is also evidence of the potential role of SWS in particular for emotional/affective wellbeing. Finan et al. (2015), for example, showed that the relationship between sleep continuity disruption and positive mood was mediated by a reduction in SWS. Furthermore, SWS has been associated with the regulation of anxiety (Ben Simon et al., 2020) and has been highlighted as essential for restoration, which may contribute to the reduction of stress and negative emotions (Cudney et al., 2022), which may in turn lead to higher positive affect. However, we only found evidence for a relationship with higher positive affect, but not with lower negative affect, so the evidence remains ambiguous. Overall, research to date has not definitively dissociated the roles of REM and SWS in emotional processing, and other sleep stages may also play a role. Ultimately, it is reasonable to assume that the interaction between different sleep stages could be particularly important (Cairney et al., 2015; Tempesta et al., 2018).

The finding that higher SE than usual was associated with lower negative affect than usual reflects the previous research reporting a negative impact of disrupted sleep on affective wellbeing (Hickman et al., 2024; Finan et al., 2017; Konjarski et al., 2018). Our study strengthens the claim that sleep continuity indicated by SE is crucial for maintaining a balanced emotional state and sleep fragmentation can impair emotional experience the following day (Konjarski et al., 2018).

Also, REM sleep's association with lower negative affect on the within-subject level is in line with research suggesting that REM could improve the processing of emotional information by creating a biological environment that depotentiates prior negative experiences and reduces emotional reactivity (Hobson et al., 1998; Konjarski et al., 2018). In particular, the SFSR theory (Walker & van der Helm, 2009) suggests that REM sleep helps consolidate declarative memory (Cunningham et al., 2014; Hu et al., 2006; Payne & Kensinger, 2010; Wiesner et al., 2015) alongside reducing associated affective tone, which may result in improved subsequent affect states. In that regard, Messman et al. (2021) found evidence for the opposite direction of the effect, i.e., higher negative affect was associated with more REM the following night.

Consistent with previous research (Hachenberger et al., 2023; Hickman et al., 2024; Lenneis et al., 2024; Werner et al., 2025), SSQ emerged as a particularly strong and consistent predictor of positive affect. For negative affect, in contrast, the evidence was rather ambiguous (ten Brink et al., 2022). In the current study, better SSQ than usual was associated with both higher positive affect and lower negative affect than usual the following morning. This finding underscores the importance of an individual's subjective perception of sleep in relation to their mood (Hachenberger et al., 2023; Lenneis et al., 2024; Werner et al., 2025), echoing prior research that emphasizes the overall importance of subjective satisfaction or evaluation for subjective health and wellbeing ratings (Kööts-Ausmees et al., 2016). The models in which SSQ predicted subsequent positive affect explained substantially more within-subject variation than all objective sleep indices combined. For negative affect, the difference between SSQ and the objective sleep indicators was less pronounced, in that the variance explained by SSQ was smaller than for positive affect which aligns with previous research (ten Brink et al., 2022).

No evidence was found to suggest that depressive or insomnia symptoms moderated the bidirectional within-subject associations between affect and sleep. It is possible that moderation effects only emerge at the severe end of the symptom spectrum. Although our sample included individuals with a wide range of clinical symptoms – such as high rates of subthreshold insomnia and moderate to severe depressive symptoms – the statistical power may have been insufficient to detect such moderating effects in a non-clinical sample where the effect sizes of these moderating effects may be rather small.

### Strengths and limitations

The study's use of objective sleep measures, derived from HRV-IBI, represents an important methodological advance (Topalidis et al., 2023a, 2023b), as it allows for unobtrusive, long-term assessment of sleep architecture. This is especially relevant given the limited availability of studies examining the within-subject associations of sleep architecture and affective wellbeing over time. In addition, the sample size in this study is considerably larger than in these previous studies (Messman et al., 2021; Zapalac et al., 2024). Also, the sample is also heterogeneous in terms of clinical symptoms (i.e., relatively high rates of subthreshold insomnia and moderate-severe depressive symptoms).

Despite the strengths of our study, there are limitations that warrant consideration. First, while the use of HRV-derived sleep staging offers advantages particularly regarding intensive longitudinal data collection, it does not capture the full complexity of sleep architecture compared to polysomnography. For instance, the distinction between NREM1 and NREM2 is not possible. However, it is unobtrusive and can be measured over multiple nights, increasing the ecological validity of findings relative to PSG. Second, the sample consisted predominantly of young adults (students, mostly female), which may limit the generalizability of the findings to other age groups. Third, while our study captured day-to-day or night-to-night variability over two weeks, longer-term studies might reveal different patterns concerning the within-subject relationship between sleep indicators and affective wellbeing by capturing a higher within-subject variability of these measures. Fourth, since we investigated the relationships between sleep and affect in our participants' daily lives, there may have been unmeasured context-specific factors such as stressful events or travel that were not taken into account in our analyses. Fifth, due to the observational nature of our study, we cannot draw any causal conclusions or infer mechanisms between the sleep and affect variables. Finally, our study focused on a typical sample of young adults who are generally healthy, albeit with a substantial proportion of participants reporting insomnia and depressive symptoms at baseline. Accordingly, we cannot draw any conclusions in this study about how sleep-affect dynamics differ in affective disorders or sleep disorders, for example, compared to healthy populations.

### Directions for future research

As mentioned in the limitations our results cannot be generalized to other age groups than young adults. Given that sleep patterns change across the lifespan, future research should examine these relationships in more diverse populations. Furthermore, future research could benefit from examining these relationships in clinical populations to better understand how sleep-affect dynamics may differ in the context of affective and sleep disorders. Methods such as photoplethysmography can also measure the interbeat intervals of the heartbeat reliably and thus may represent an even less obtrusive method of measuring cardiac activity from which sleep parameters could be derived compared to chest straps, as used in our study. Future research should also investigate the sleep-affect relationship – particularly with regard to sleep architecture – in the context of intervention studies (e.g., ambulatory sleep/bedtime restriction) or experimental designs involving sleep phase suppression (e.g., Fehér et al., 2023).

## Conclusion

The findings of this study suggest that sleep itself may have a profound effect on subsequent affective wellbeing. The results underscore the importance of focusing sleep health, especially in therapeutic contexts, to enhance affect and reduce negative emotions. It may be indicated to put more emphasis on non-restorative sleep and on programs aiming to improve sleep. Overall, these insights could inform more effective treatments for improving both mental health and sleep.

## Funding

This research received no external funding.

## Ethics

Both studies that we report on were approved by the Ethics Committee of Bielefeld University (reference number 2023–078 and 2023–195).

## CRediT authorship contribution statement

**Justin Hachenberger:** Conceptualization, Data curation, Formal analysis, Investigation, Methodology, Project administration, Writing – original draft, Writing – review & editing. **Maia ten Brink:** Conceptualization, Formal analysis, Writing – review & editing. **Denny Kerkhoff:** Investigation, Writing – review & editing. **Sebastian Baron:** Software, Writing – review & editing. **Manuel Schabus:** Conceptualization, Software, Writing – review & editing. **Sakari Lemola:** Conceptualization, Methodology, Project administration, Supervision, Writing – review & editing.

## Declaration of competing interest

The authors declare that they have no known competing financial interests or personal relationships that could have appeared to influence the work reported in

Manuel Schabus is CSO for the sleep² App and co-founder of the Nukkuaa GmbH which developed the MCNN model for HRV-based sleep classification. Otherwise, the authors declare that they have no known competing financial interests or personal relationships that could have appeared to influence the work reported in this paper.

## Data Availability

The data and analysis code that support the findings of this study are available from the corresponding author, upon request.

## Appendix Supplementary materials

Image, application 1
