# Supplementary material for: Exploring the bidirectional within-subject relationship between sleep and affective wellbeing: Insights from an intensive longitudinal study
Source: Int J Clin Health Psychol. 2025 Nov 13;25(4):100648. doi: 10.1016/j.ijchp.2025.100648 (PMC12662039; doi:10.1016/j.ijchp.2025.100648)
Supplement: Supplementary file 1 [file mmc1.pdf]

Supplementary Material

Exploring the Bidirectional Within-Subject Relationship Between Sleep and Affective Wellbeing: Insights from an Intensive Longitudinal Study

Justin Hachenberger[*,1], Maia ten Brink[2], Denny Kerkhoff[1], Sebastian Baron[3], Manuel Schabus[4] and Sakari Lemola[1]

[1] Department of Psychology, Faculty of Psychology and Sports Science, Bielefeld University, Germany

[2] Columbia University Irving Medical Center, Center for Behavioral Cardiovascular Health, Department of Medicine, New York, NY, United States

[3] Department of Artificial Intelligence and Human Interfaces (AIHI), Paris-Lodron University of Salzburg, Salzburg, Austria

[4] Laboratory for Sleep, Cognition and Consciousness Research, Department of Psychology, Centre for Cognitive Neuroscience Salzburg (CCNS), Paris-Lodron University of Salzburg, Salzburg, Austria

Supplement 1

Equations for the Multilevel Models Used for the Statistical Analyses

Research Question 1

Multilevel models testing Research Question 1 were defined by the following equations:

Level-1:

$$Y_{ij} = \beta_{0j} + \beta_{1j} * Positive/Negative\ Affect_{ij} + Covariates + r_{ij}$$

Level-2:

$$\beta_{0j} = \gamma_{00} + u_{0j}$$

$$\beta_{1j} = \gamma_{10} + u_{1j}$$

The within-subject effects were estimated at level 1. The subscript j refers to the participant and subscript i to the time of measurement. The sleep metrics (TST, SE, SOL, WASO, NOA, SWS, REM, NSS, SSQ; separate models for each metric were calculated) at time i in participant j are represented by Y_{ij}. The intercept and the effects of positive/negative affect (separate models) are represented by β at level 1. The residuals are represented by r_{ij}. At level 2 we included a random intercept and a random slope for positive/negative affect. u_{0j} represents the variation of participants' intercept around the mean intercept (γ_{00}). u_{1j} represent the variation of participants' individual slopes for positive/negative affect around the respective overall mean slope (γ_{10}).

Research Question 2

Multilevel models testing Research Question 2 were defined by the following equations:

Level-1:

$$Y_{ij} = \beta_{0j} + \beta_{1j} * TST_{ij} + \beta_{2j} * SE_{ij} + \beta_{3j} * SOL_{ij} + \beta_{4j} * WASO_{ij} + \beta_{5j} * NOA_{ij} + \beta_{6j} * SWS_{ij} + \beta_{7j} * REM_{ij} + \beta_{8j} * NSS_{ij} + Covariates + r_{ij}$$

Level-2:

$$\beta_{0j} = \gamma_{00} + u_{0j}$$

$$\beta_{1j} = \gamma_{10} + u_{1j}$$

$$\beta_{2j} = \gamma_{20} + u_{2j}$$

$$\beta_{3j} = \gamma_{30} + u_{3j}$$

$$\beta_{4j} = \gamma_{40} + u_{4j}$$

$$\beta_{5j} = \gamma_{50} + u_{5j}$$

$$\beta_{6j} = \gamma_{60} + u_{6j}$$

$$\beta_{7j} = \gamma_{70} + u_{7j}$$

$$\beta_{8j} = \gamma_{80} + u_{8j}$$

The within-subject effects were estimated at level 1. The subscript j refers to the participant and subscript i to the time of measurement. The positive/negative affect (in separate models) at time i in participant j are represented by Y_{ij}. The intercept and the effects of the sleep indicators are represented by β at level 1. The residuals are represented by r_{ij}. At level 2 we included a random intercept and a random slope for each sleep predictor. u_{0j} represents the variation of participants' intercept around the mean intercept (γ_{00}). u_{1j} to u_{8j}represent the variation of participants' individual slopes for the respective sleep indicator around the respective overall mean slope (γ_{10} to γ_{80}). After the initial full model, non-significant sleep predictors are iteratively removed until only significant predictors remain.

SSQ was tested separately:

Level-1:

$$Y_{ij} = \beta_{0j} + \beta_{1j} * SSQ_{ij} + Covariates + r_{ij}$$

Level-2:

$$\beta_{0j} = \gamma_{00} + u_{0j}$$

$$\beta_{1j} = \gamma_{10} + u_{1j}$$

The within-subject effects were estimated at level 1. The subscript j refers to the participant and subscript i to the time of measurement. The positive/negative affect (in separate models) at time i in participant j are represented by Y_{ij}. The intercept and the effects of SSQ are represented by β at level 1. The residuals are represented by r_{ij}. At level 2 we included a random intercept and a random slope for SSQ. u_{0j} represents the variation of participants' intercept around the mean intercept (γ_{00}). u_{1j} represent the variation of participants' individual slopes for SSQ around the respective overall mean slope (γ_{10}).

Research Question 3

To address RQ3, the models described for RQ1 and RQ2 were extended by including the time-invariant (between-level) depressive symptoms and insomnia symptoms as moderators and their cross-level interactions with the level-1 predictors.

Multilevel models extending the analyses for RQ1 by including a cross-level interaction (depressive and insomnia symptoms) were defined by the following equations:

Level-1:

$$Y_{ij} = \beta_{0j} + \beta_{1j} * Positive/Negative\ Affect_{ij} + Covariates + r_{ij}$$

Level-2:

$$\beta_{0j} = \gamma_{00} + u_{0j}$$

$$\beta_{1j} = \gamma_{10} + \gamma_{11} * Depressive/Insomnia\ Symptoms_j + u_{1j}$$

The within-subject effects were estimated at level 1. The subscript j refers to the participant and subscript i to the time of measurement. The sleep metrics (TST, SE, SOL, WASO, NOA, SWS, REM, NSS, SSQ; separate models for each metric were calculated) at time i in participant j are represented by Y_{ij}. The intercept and the effects of positive/negative affect (separate models) are represented by β at level 1. The residuals are represented by r_{ij}. At level 2 we included a random intercept and a random slope for positive/negative affect. u_{0j} represents the variation of participants' intercept around the mean intercept (γ_{00}). u_{1j} represent the variation of participants' individual slopes for positive/negative affect around the respective overall mean slope (γ_{10}). γ_{11} represents the coefficient for the cross-level interaction (depressive or insomnia symptoms).

Multilevel models extending the analyses for RQ2 by including a cross-level interaction (depressive and insomnia symptoms) were defined by the following equations:

Level-1:

$$Y_{ij} = \beta_{0j} + \beta_{1j} * TST_{ij} + \beta_{2j} * SE_{ij} + \beta_{3j} * SOL_{ij} + \beta_{4j} * WASO_{ij} + \beta_{5j} * NOA_{ij} + \beta_{6j} * SWS_{ij} + \beta_{7j} * REM_{ij} + \beta_{8j} * NSS_{ij} + Covariates + r_{ij}$$

Level-2:

$$\beta_{0j} = \gamma_{00} + u_{0j}$$

$$\beta_{1j} = \gamma_{10} + \gamma_{11} * Depressive/Insomnia\ Symptoms_j + u_{1j}$$

$$\beta_{2j} = \gamma_{20} + \gamma_{21} * Depressive/Insomnia\ Symptoms_j + u_{2j}$$

$$\beta_{3j} = \gamma_{30} + \gamma_{31} * Depressive/Insomnia\ Symptoms_j + u_{3j}$$

$$\beta_{4j} = \gamma_{40} + \gamma_{41} * Depressive/Insomnia\ Symptoms_j + u_{4j}$$

$$\beta_{5j} = \gamma_{50} + \gamma_{51} * Depressive/Insomnia\ Symptoms_j + u_{5j}$$

$$\beta_{6j} = \gamma_{60} + \gamma_{61} * Depressive/Insomnia\ Symptoms_j + u_{6j}$$

$$\beta_{7j} = \gamma_{70} + \gamma_{71} * Depressive/Insomnia\ Symptoms_j + u_{7j}$$

$$\beta_{8j} = \gamma_{80} + \gamma_{81} * Depressive/Insomnia\ Symptoms_j + u_{8j}$$

The within-subject effects were estimated at level 1. The subscript j refers to the participant and subscript i to the time of measurement. The positive/negative affect (in separate models) at time i in participant j are represented by Y_{ij}. The intercept and the effects of the sleep indicators are represented by β at level 1. The residuals are represented by r_{ij}. At level 2 we included a random intercept and a random slope for each sleep predictor. u_{0j} represents the variation of participants' intercept around the mean intercept (γ_{00}). u_{1j} to u_{8j}represent the variation of participants'

individual slopes for the respective sleep indicator around the respective overall mean slope (γ_{10} to γ_{80}). After the initial full model, non-significant sleep predictors are iteratively removed until only significant predictors remain. γ_{11} to γ_{81} represent the coefficient for the cross-level interaction (depressive or insomnia symptoms).

SSQ was tested separately:

Level-1:

$$Y_{ij} = \beta_{0j} + \beta_{1j} * SSQ_{ij} + Covariates + r_{ij}$$

Level-2:

$$\beta_{0j} = \gamma_{00} + u_{0j}$$

$$\beta_{1j} = \gamma_{10} + \gamma_{11} * Depressive/Insomnia\ Symptoms_j + u_{1j}$$

The within-subject effects were estimated at level 1. The subscript j refers to the participant and subscript i to the time of measurement. The positive/negative affect (in separate models) at time i in participant j are represented by Y_{ij}. The intercept and the effects of SSQ are represented by β at level 1. The residuals are represented by r_{ij}. At level 2 we included a random intercept and a random slope for SSQ. u_{0j} represents the variation of participants' intercept around the mean intercept (γ_{00}). u_{1j} represent the variation of participants' individual slopes for SSQ around the respective overall mean slope (γ_{10}). γ_{11} represents the coefficient for the cross-level interaction (depressive or insomnia symptoms).
